# Is Increased Intracellular Calcium in Red Blood Cells a Common Component in the Molecular Mechanism Causing Anemia?

**DOI:** 10.3389/fphys.2017.00673

**Published:** 2017-09-06

**Authors:** Laura Hertz, Rick Huisjes, Esther Llaudet-Planas, Polina Petkova-Kirova, Asya Makhro, Jens G. Danielczok, Stephane Egee, Maria del Mar Mañú-Pereira, Richard van Wijk, Joan-Lluis Vives Corrons, Anna Bogdanova, Lars Kaestner

**Affiliations:** ^1^Research Centre for Molecular Imaging and Screening, Medical School, Saarland University Homburg, Germany; ^2^Department of Clinical Chemistry and Haematology, University Medical Center Utrecht Utrecht, Netherlands; ^3^Red Blood Cell Defects and Hematopoietic Disorders Unit, Josep Carreras Leukaemia Research Institute Barcelona, Spain; ^4^Red Blood Cell Research Group, Institute of Veterinary Physiology, Vetsuisse Faculty and the Zurich Center for Integrative Human Physiology (ZIHP), University of Zurich Zurich, Switzerland; ^5^Centre National de la Recherche Scientifique, UMR 8227 Comparative Erythrocyte's Physiology Roscoff, France; ^6^Université Pierre et Marie Curie, Sorbonne Universités Roscoff, France; ^7^Laboratoire d'Excellence GR-Ex Roscoff, France; ^8^Theoretical Medicine and Biosciences, Saarland University Homburg, Germany; ^9^Experimental Physics, Saarland University Saarbruecken, Germany

**Keywords:** rare anemia, erythrocyte, calcium homeostasis, channelopathies, live cell imaging, spherocytosis, xerocytosis

## Abstract

For many hereditary disorders, although the underlying genetic mutation may be known, the molecular mechanism leading to hemolytic anemia is still unclear and needs further investigation. Previous studies revealed an increased intracellular Ca^2+^ in red blood cells (RBCs) from patients with sickle cell disease, thalassemia, or Gardos channelopathy. Therefore we analyzed RBCs' Ca^2+^ content from 35 patients with different types of anemia (16 patients with hereditary spherocytosis, 11 patients with hereditary xerocytosis, 5 patients with enzymopathies, and 3 patients with hemolytic anemia of unknown cause). Intracellular Ca^2+^ in RBCs was measured by fluorescence microscopy using the fluorescent Ca^2+^ indicator Fluo-4 and subsequent single cell analysis. We found that in RBCs from patients with hereditary spherocytosis and hereditary xerocytosis the intracellular Ca^2+^ levels were significantly increased compared to healthy control samples. For enzymopathies and hemolytic anemia of unknown cause the intracellular Ca^2+^ levels in RBCs were not significantly different. These results lead us to the hypothesis that increased Ca^2+^ levels in RBCs are a shared component in the mechanism causing an accelerated clearance of RBCs from the blood stream in channelopathies such as hereditary xerocytosis and in diseases involving defects of cytoskeletal components like hereditary spherocytosis. Future drug developments should benefit from targeting Ca^2+^ entry mediating molecular players leading to better therapies for patients.

## Introduction

Anemia, defined as a hemoglobin concentration <11–13 g/dl, based on gender and age, affects 1.6 billion people worldwide (McLean et al., 2009). About 10% of these individuals are affected by rare anemias. This disease group includes ~90 different types of red blood cell (RBC) diseases, of which 80% are hereditary or congenital in nature. As the pathophysiology of most of these rare anemias is poorly understood, the appropriate treatment is often ineffective or even lacking.

Anemia in general has three major causes: blood loss, insufficient hematopoiesis, or facilitated removal of RBCs from the blood stream (Ossendorf, 2003). In hemolytic anemias the RBC premature clearance and shortened lifespan of RBCs are not compensated by enhanced RBC production giving rise to anemia (Dhaliwal et al., 2004). Many types of anemia can be assigned to mutations in a single protein. However, it is still unclear how these mutations transfer into an increased clearance of RBCs and what defines heterogeneity in disease severity. A prominent example is sickle cell disease. Described as a molecular disease as early as 1949 (Pauling et al., 1949), sickle cell disease is caused by a single point mutation in the ß-globin gene (Ingram, 1958). The mutated hemoglobin variant, HbS, is prone to polymerization and formation of HbS aggregates, that are even more likely to occur upon dehydration of RBCs (Layton and Nagel, 2010). Dehydration is largely mediated by high intracellular Ca^2+^, subsequent activation of Gardos Channels and loss of K^+^ (Lew et al., 2002). Ca^2+^ uptake in sickle RBCs is abnormally high, and not always compensated by Ca^2+^ extrusion by the Ca^2+^ pumps resulting in an elevated intracellular Ca^2+^ content (Eaton et al., 1973; Tiffert et al., 2003). This increase in the Ca^2+^ content is attributed to highly abundant hyperactive NMDA receptors in membranes of patients' RBCs. Inhibition of Ca^2+^ uptake via these receptors could prevent dehydration and sickling of RBCs *in vitro* (Hänggi et al., 2014; Bogdanova et al., 2015). However, it remains elusive, how the mutation in the hemoglobin “causes” the increased Ca^2+^-influx. The fact that vaso-occlusive crises in sickle cell disease patients occur sporadically (Rieber et al., 1977) points to a rather indirect connection. Increased intracellular Ca^2+^ levels were also found in RBCs from, e.g., beta thalassemia patients (Bookchin et al., 1988) or patients with Gardos channelopathy (Fermo et al., 2017). It is known that Ca^2+^ overload triggers several downstream events in RBCs (Bogdanova et al., 2013). One important effect is the impairment of the cytoskeletal stability, e.g., through activation of calpain and subsequent cleavage of membrane associated proteins (Inomata et al., 1993; Salamino et al., 1993). The activation of calmodulin and its interaction with the band 4.1R protein has been shown to decrease the affinity of 4.1R for its cytoskeletal interaction partners actin and spectrin and thereby loosening the cytoskeletal structure (Jarret and Kyte, 1979; Nunomura and Takakuwa, 2006). A decreased RBC volume is resulting from the Ca^2+^ dependent opening of the Gardos channel, which leads to loss of K^+^, Cl^−^ and water (Gardos, 1958). Furthermore, increased Ca^2+^ levels lead to the disruption of the asymmetrical distribution of phospholipids in the plasma membrane. Phosphatidylserine, a lipid exclusively present in the inner leaflet of the membrane, becomes exposed on the outer membrane by activation of the scramblase and simultaneous inhibition of the flippase (Verkleij et al., 1973; Bitbol et al., 1987; Bassé et al., 1996; Woon et al., 1999). All these described changes in the cell physiology as well as the increased Ca^2+^ are signs of senescence (sometimes referred to as eryptosis) and prime the cells for clearance from the blood stream (Lutz and Bogdanova, 2013). A substantial increase in intracellular Ca^2+^ also increases the osmotic fragility with no strict correlation to cell volume and largely before cells reach spherocytic hemolysis volume (Cueff et al., 2010). This might be an additional mechanism of a decrease in RBC number associated to an elevated Ca^2+^concentration. To what extent a Ca^2+^ induced increased vesiculation (Nguyen et al., 2011; Alaarg et al., 2013) may alter the RBC clearance is still unknown.

Here we aim to investigate if elevated intracellular Ca^2+^ levels are a general feature in the pathophysiology of hemolytic anemia and such provides a mechanistic link for an increased clearance of RBCs resulting in anemia of hemolytic patients.

## Materials and methods

### Participants

Patients diagnosed with different types of anemia were enrolled in the study after signed informed consent. Patient data were handled anonymously as outlined in the ethics applications. These applications were approved by the Medical Ethical Research Board (MERB) of the University Medical Center Utrecht, the Netherlands, (UMCU) under reference code 15/426M “Disturbed ion homeostasis in hereditary hemolytic anemia” and also by the Ethical Committee of Clinical Investigations of Hospital Clinic, Spain, (IDIBAPS) under the reference code 2013/8436. Exclusion criteria were erythrocyte transfusion in the past 90 days, age below 3 years and/or body weight lower than 18 kg. Blood from healthy control donors was anonymously obtained using the approved medical ethical protocol of 07/125 Mini Donor Dienst, also approved by the MERB of UMCU. The blood of the patient and the healthy donor anti-coagulated in lithium-heparin was shipped overnight from the University Medical Center Utrecht (Utrecht, The Netherlands) and from Institut d'Investigacions Biomèdiques August Pi i Sunyer/Hospital Clínic de Barcelona (Barcelona, Spain) to Saarland University (Homburg, Germany). All patients included in this study were genetically screened for mutations by next-generation sequencing and diagnosed with the following types of anemia: 16 patients included in this study were diagnosed with hereditary spherocytosis using golden standard techniques (EMA-binding, osmotic gradient ektacytometry and osmotic fragility test). Moreover, these 16 patients were screened for mutations by next-generation sequencing: 7 patients had mutations in *ANK1*, 4 patients had mutations in *SPTA1*, 3 patients in *SPTB*, and 2 patients showed mutations in *SLC4A1*). Eleven patients were diagnosed with hereditary xerocytosis (due to mutations in *PIEZO1*), 5 patients had enzymatic disorders (3 patients with glucose-6-phosphate dehydrogenase deficiency, 1 patient with glutamate-cysteine ligase deficiency, 1 patient with glutathione reductase deficiency) and 3 patients suffered from hemolytic anemia of unknown cause.

### Calcium imaging

Ca^2+^ imaging experiments were carried out with RBCs from 35 patient blood samples and 25 healthy transportation controls. Intracellular Ca^2+^ was measured from single cells as Fluo-4 (Thermo Fisher Scientific, Waltham, MA, USA) based fluorescence intensity as described before (Wang et al., 2014).

### Data analysis

Analysis of the fluorescence images was performed in ImageJ (Wayne Rasband, National Institutes of Health) and further processing of the data was done using Matlab (Mathworks, Natick, MA, USA) and GraphPad Prism (GraphPad Software Inc., La Jolla, CA, USA). For each patient and control at least 200 individual cells were analyzed. The mean fluorescence intensity values were plotted as box-and-whiskers. Boxes show median and 25th to 75th percentiles, whiskers are drawn down to 10th and up to 90th percentile. For cell-based analysis of single patients and controls, significance was checked using the Mann–Whitney test.

For patient-based analysis, patients were grouped in hereditary spherocytosis, hereditary xerocytosis, enzymopathies, and hemolytic anemia of unknown cause. As a statistical basis we used the median fluorescence intensity value from the single cell analysis for each patient and control sample. Intensity values from controls were normalized to their mean value, whereas patients were normalized to the corresponding shipping control. Significance was tested on not normalized raw data using the paired *t*-test when data showed a normal distribution (D'Agostino-Pearson normality test) otherwise with the Wilcoxon signed-rank test.

## Results

**Figure 1:**
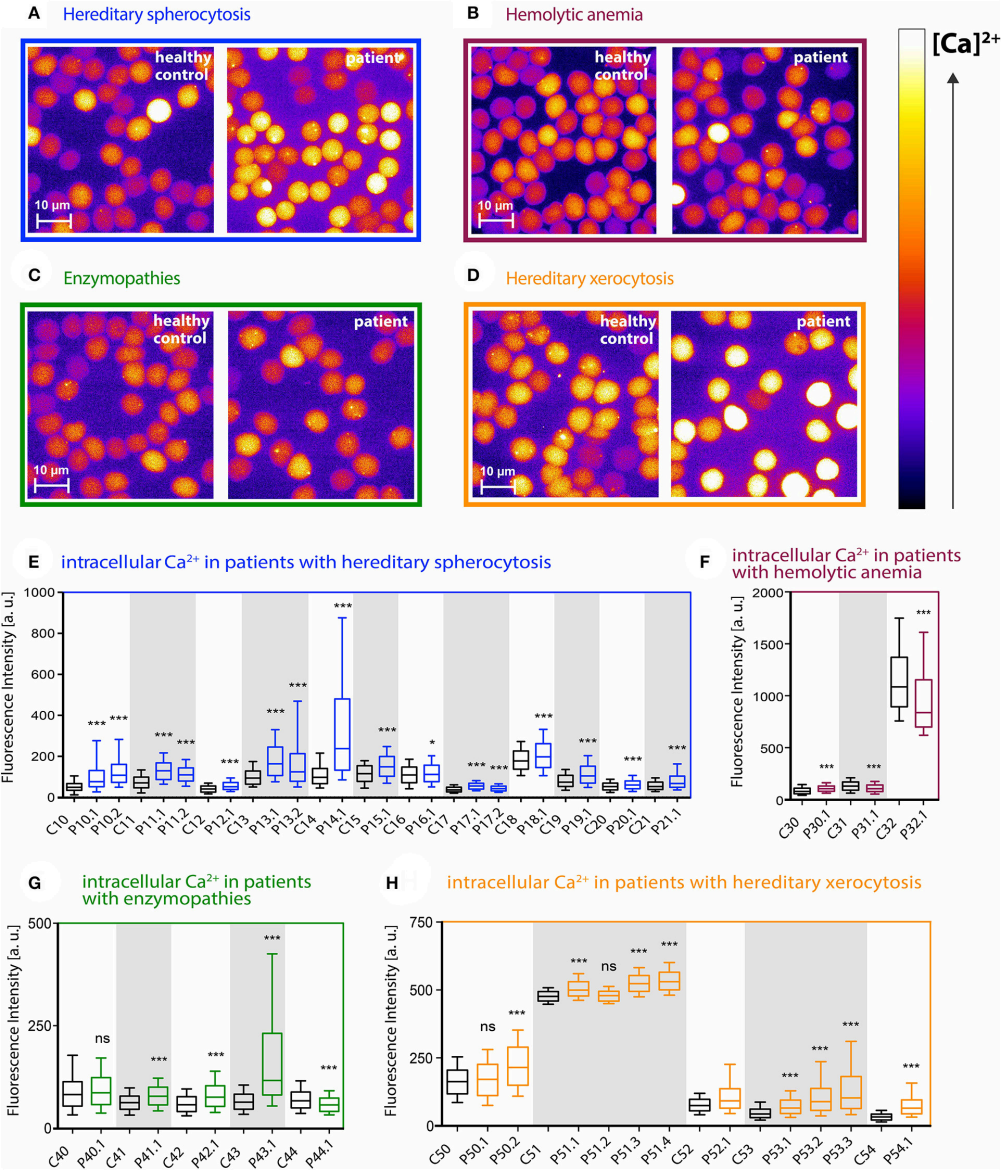
Intracellular Ca^2+^ content in RBCs from patients with different types of hemolytic anemia. **(A–D)** Representative fluorescence images of Fluo-4 loaded RBCs from control and patient blood samples. Relative intracellular Ca^2+^ concentration is scaled from black (lowest) to white (highest). Patients were grouped according to their disease diagnosis: hereditary spherocytosis (blue), hemolytic anemia (purple), enzymopathies (green), hereditary xerocytosis (orange). **(E–H)** Statistical analysis of the mean Fluo-4 intensity values in arbitrary units from single cells for each group of patients and the corresponding control samples. Controls and patients that belong to the same shipment are highlighted with a white or gray background. Controls are displayed as black boxes, patients are colored in blue (hereditary spherocytosis), purple (unknown hemolytic anemia), green (enzymopathies), and orange (hereditary xerocytosis). Whiskers indicate the range between 10th and 90th percentiles. There is a huge variation in the intensity-correlated Ca^2+^ concentrations already within the control group of healthy donors. Significance was tested between each patient and the according shipping control sample (always the next control sample on left side) using the Mann–Whitney test (ns denotes *p* > 0.5, ^*^p ≤ 0.5, ^***^p ≤ 0.01).

In this study, 35 patients with different types of hemolytic anemia were analyzed. Patients were grouped according to their disease into 4 subgroups: hereditary spherocytosis patients, hereditary xerocytosis patients, patients with enzymopathies and patients with unknown hemolytic anemia. For each subgroup, representative fluorescence images of Fluo-4 loaded RBCs from patients and corresponding transportation controls of healthy donors are depicted in Figures 1A–D.

Figures 1E–H show the cell-based statistical analysis for each patient and the corresponding control. Fluo-4 fluorescence intensity from single cells was measured and plotted. There was a huge variation in the intensity-correlated Ca^2+^ concentrations when comparing the samples taken altogether. However, when we compared samples within a particular shipment (identical transportation conditions), the Ca^2+^ concentrations were in a similar/comparable range. Therefore we always compared patients exclusively to their corresponding shipping controls.

In all 16 cases of hereditary spherocytosis we found that intracellular Ca^2+^ levels in RBCs were significantly elevated in patients compared to the corresponding healthy shipping control samples (Figure 1E). In hereditary xerocytosis 9 out of 11 patients showed increased Ca^2+^ levels, whereas for the other two patients no significant differences could be detected (Figure 1H, P50.1 and P51.2).

The group of unknown hemolytic anemia patients gave a more heterogeneous picture. The Ca^2+^ content in one patient was significantly increased, whereas the other two patients had significantly lower Ca^2+^ levels than the shipping control (Figure 1F, P30.1 vs. P31.1 and P32.1). The situation was similar for the enzymopathies: three patients (Figure 1G, P41.1, P42.1, P43.1) had an increased intracellular Ca^2+^ content, one was significantly lower and one patient showed no significant difference compared to its control (Figure 1G, P44.1 and P40.1, respectively).

**Figure 2:**
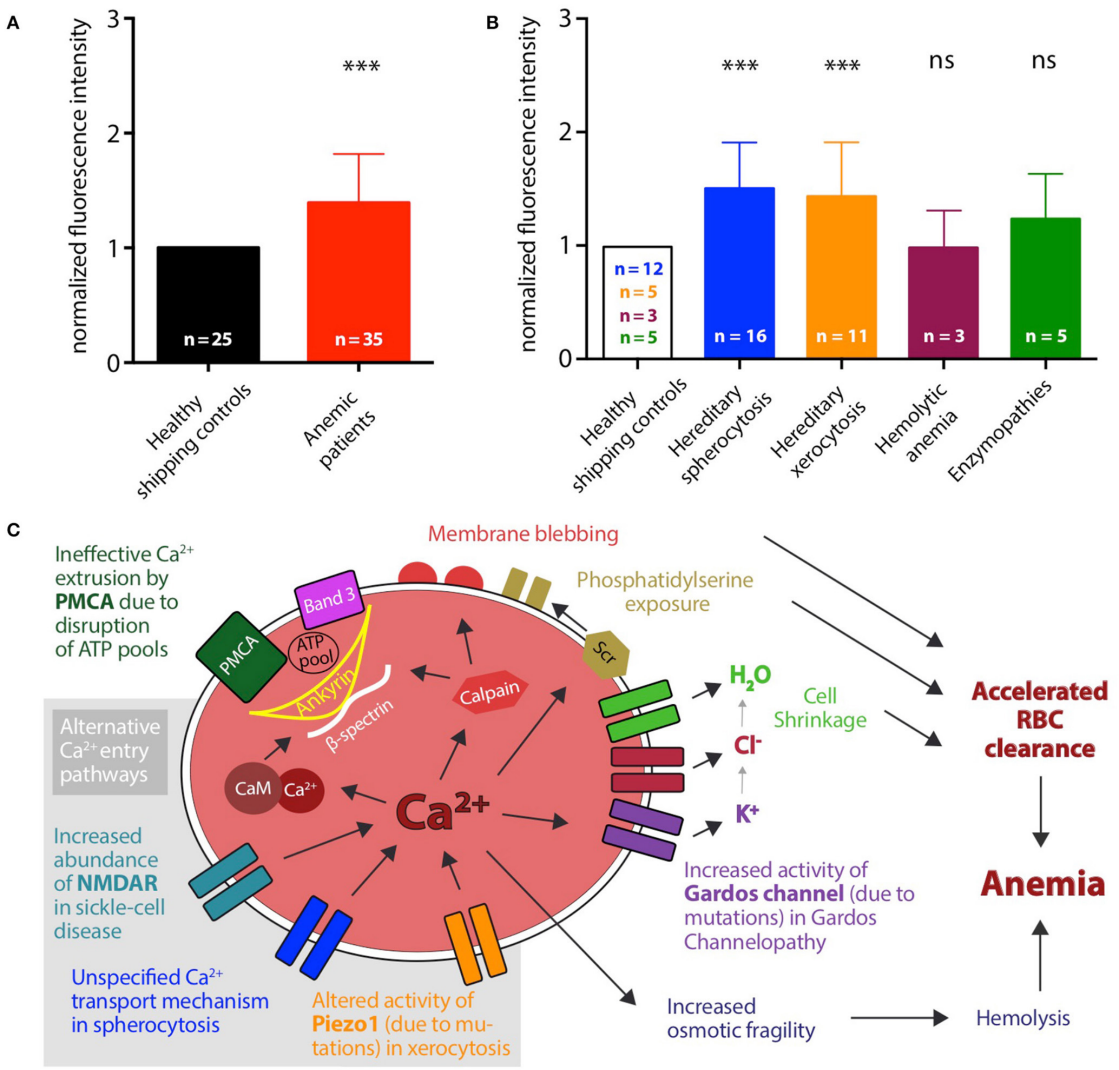
Patient-based statistical analysis of intracellular Ca^2+^ levels and proposed mechanisms. **(A)** Statistical analysis of normalized median fluorescence intensity values for all 35 patients (red bar) and 25 healthy shipping controls (black bar). Significance was tested using the Wilcoxon signed-rank test (^***^p ≤ 0.01). **(B)** Statistical analysis of normalized median fluorescence intensity values for controls and patients grouped by disease. Number of control samples differs for each disease group. Hereditary spherocytosis (blue): 16 patients and 12 controls, hereditary xerocytosis (orange): 12 patients and 5 controls, hemolytic anemia (purple): 3 patients and 3 controls, enzymopathies (green): 5 patients and 5 controls. Significance was tested using the paired *t*-test, when data showed a Gaussian distribution (D'Agostino-Pearson normality test), otherwise using the Wilcoxon signed-rank test (ns denotes *p* > 0.5, ^***^p ≤ 0.01). **(C)** Proposed mechanisms leading to increased intracellular Ca^2+^ levels in diseased RBCs and accordingly to accelerated clearance of cells from the blood stream. Alternative or cumulating Ca^2+^ entry pathways are highlighted with gray background: increased abundance of NMDA-receptors (NMDAR), e.g., in sickle cell disease, altered activity of Piezo1, e.g., in hereditary xerocytosis, increased activity of Gardos Channel, e.g., in Gardos Channelopathy, or unspecified Ca^2+^ transport mechanisms. Additionally, ineffective extrusion of Ca^2+^ due to disruption of ATP pools fueling the plasma membrane Ca^2+^ ATPase (PMCA) can contribute. Several downstream processes follow Ca^2+^ overload in RBCs, e.g.: activation of calmodulin by formation of the Ca^2+^-calmodulin complex (Ca-CaM) and activation of calpain, thereby loosening the cytoskeletal structure; activation of the scramblase (Scr) leading to exposure of phosphatidylserine on the outer leaflet of the membrane; activation of the Gardos channel followed by the efflux of K^+^, Cl^−^ and H_2_O and consecutive cell shrinkage.

The patient-based statistical analysis of the Ca^2+^ concentrations is depicted in Figure 2A and shows the paired and normalized analysis of all patients in comparison to all controls. Ca^2+^ levels in hemolytic anemia patients show a highly significant increase. Figure 2B shows the same analysis for subgroups of patients. The groups with hereditary spherocytosis and hereditary xerocytosis patients have significantly increased levels of intracellular Ca^2+^, compared to the controls. Patients with enzymopathies depict an increase in the intracellular Ca^2+^ concentration but fail to reach significant changes. For patients with hemolytic anemia of unknown cause intracellular Ca^2+^ levels appear to be heterogeneous, because it is a non-systematic group composition.

## Discussion

### Ca^2+^ overload

In this study we monitored the intracellular Ca^2+^ levels in RBCs from patients with several types of hemolytic anemia. We found that in the analyzed cases of membranopathies, hereditary spherocytosis and hereditary xerocytosis, intracellular Ca^2+^ is significantly increased. Figure 2C depicts the proposed mechanism. Starting with the previously described sickle cell disease (compare Introduction) an increased number of NMDA receptors were identified to cause the abnormal uptake of Ca^2+^ (Hänggi et al., 2014). *In vitro*, this increase in Ca^2+^ can be reduced by NMDA blockers like Memantine and a pilot clinical trial investigating the effect of this drug on sickle cell disease patients is closed and the statistical analysis is currently on-going (Bogdanova et al., 2017). For other types of anemia the Ca^2+^ entry pathways are rather unclear. In the Gardos channelopathy it is suggested that Ca^2+^ enters via the mechanical activated Piezo1 channel (Fermo et al., 2017). We found several different mutations for the *PIEZO1* gene in the xerocytosis patients included in our study. A further characterization of how these mutations affect the physiology of the channel is still ongoing, but data in the literature show that six dehydrated hereditary stomatocytosis-causing mutations in the Piezo1 channel with five of them in the C-terminal 1/5 of the protein, result in a slowing of the inactivation kinetics of the channel and thus to a more active channel (Albuisson et al., 2013). However, independent of Ca^2+^ entry pathways also an impairment of Ca^2+^ extrusion (of residual Ca^2+^ influx) needs to be considered as an important factor for the Ca^2+^ homeostasis in RBCs. The plasma membrane Ca^2+^ ATPase (PMCA) is the only known active extrusion pathway for Ca^2+^ in RBCs and its transportation capacity is limited by the availability of ATP (Schatzmann, 1966; Pasini et al., 2006). It has been reported that Piezo1 can regulate the ATP release from human RBCs and that mutations in the channel can alter the released amount of ATP (Cinar et al., 2015). Therefore it is also possible that the detected Ca^2+^ overload in RBCs from xerocytosis patients is due to ATP depletion and ineffective Ca^2+^ extrusion. This would imply that glycolytic enzyme defects of the RBC, generally considered to lead to decreased ATP levels, would result in Ca^2+^ overload. The ATP needed to fuel the pump is trapped in membrane-associated complexes (ATP pools). Among others, identified components of these complexes are Band 3, ankyrin, and β-spectrin (Chu et al., 2012). Mutations in these proteins are found in the spherocytosis patients included in this study. If these mutations lead to a disruption of the complexes serving as ATP pools for the Ca^2+^ pump, the extrusion of Ca^2+^ will also be significantly reduced. Likewise, the general cytoskeleton stability is impaired in RBCs from spherocytosis patients. Changes in the mechanical stability of the cells may result in an activation of mechanosensitive channels, like Piezo1, again leading to an increase in intracellular Ca^2+^. Both components (increased Ca^2+^ leak and decreased Ca^2+^ extrusion) could happen independent of each other or even mutually reinforce. We can exclude that the different values in fluorescence intensity are due to changes in cell volume: In a different study we tested volume changes, e.g., by osmotic swelling, and co-stained RBCs with Calcein Red-Orange, a dye insensitive to the Ca^2+^ concentration and intensity changes were negligible compared to intensity changes occurring in measurements probing the RBCs Ca^2+^ concentration (Danielczok et al., 2015). The delicate balance between Ca^2+^ entry and exit is likely to be influenced by mechanical stress. During their journey in the blood stream, RBCs experience hundreds squeezes when passing small capillaries and the spleen. To perform such experiments in artificial circulation systems *in vitro* became recently available based on microfluidic devices (Brust et al., 2014; Danielczok et al., 2015; Picot et al., 2015) and should be a future experimental focus.

The results we report do have a therapeutic impact: they illustrate that disease specific pharmacological targets should be upstream of the increase in intracellular Ca^2+^ to avoid all Ca^2+^ related effects that accumulate the processes facilitating RBC clearance as outlined in Figure 2C. In contrast pharmacological targets downstream of the action of intracellular Ca^2+^ may fail to address the major symptoms of the disease as it was shown for the Gardos channel inhibitor Senicapoc. In sickle cell disease patients it failed to improve acute vaso-occlusive crises (Ataga et al., 2011).

### Blood shipment

Having a critical look at our data from Figures 1E–H it is evident that the transportation process has a huge impact on the measured intracellular Ca^2+^ content in RBCs. Differences (within controls) are mainly not due to different transportation times (which were similar for all shipments) but to different transportation conditions, like temperature, vibration intensity and duration or other shipment related parameters that are out of our control. We choose Heparin as an anticoagulant because it does not interfere with the extracellular Ca^2+^. Other standard anticoagulants like CPDA or EDTA bind the external Ca^2+^, making internal Ca^2+^ measurements unreliable (Makhro et al., 2016). In the mentioned study transportation was simulated under laboratory conditions, which shows better results than the inconsistency of control samples within the present experiments (Figures 1E–H). In addition we do not know if intracellular Ca^2+^ levels during transportation are more severely influenced in patient RBCs compared to healthy controls, which presents an uncertainty of this study. Therefore future studies require investigations without cellular convolution by the shipment process, i.e., either a travel of the patient to specialized laboratories for single cell investigations or the establishment of such specialized mobile laboratories.

## Conclusion

Considering our Ca^2+^ data and the results from studies on sickle cells and RBCs from patients with Gardos channelopathies, there is strong evidence that the Ca^2+^ overload in RBCs contributes to their accelerated clearance from the circulation and is a common part of the molecular mechanism in these types of anemia. The exact molecular regulation of the Ca^2+^ entry pathways requires further investigations. Future drug developments should benefit from targeting Ca^2+^ entry mediating molecular players, as, e.g., Memantine for the treatment of sickle cell disease patients (Bogdanova et al., 2017), leading to better therapies for patients.

## Author contributions

LK, AB, RvW, JV defined the study and planed the experiments. LH, RH, EL, PP, JD, AM, MdM performed the acquisition and analysis. LH, SE, and LK interpreted the data. LH and LK drafted the manuscript. AB, RvW, JV, RH, SE, EL, PP, JD, AM, MdM critically revised the manuscript. All authors approved the final version of the manuscript.

### Conflict of interest statement

The authors declare that the research was conducted in the absence of any commercial or financial relationships that could be construed as a potential conflict of interest.
